# Brain Degeneration in Synucleinopathies Based on Analysis of Cognition and Other Nonmotor Features: A Multimodal Imaging Study

**DOI:** 10.3390/biomedicines11020573

**Published:** 2023-02-15

**Authors:** Olaia Lucas-Jiménez, Naroa Ibarretxe-Bilbao, Ibai Diez, Javier Peña, Beatriz Tijero, Marta Galdós, Ane Murueta-Goyena, Rocío Del Pino, Marian Acera, Juan Carlos Gómez-Esteban, Iñigo Gabilondo, Natalia Ojeda

**Affiliations:** 1 Department of Psychology, Faculty of Health Sciences, University of Deusto, 48007 Bilbao, Spain; 2 Gordon Center for Medical Imaging, Department of Radiology, Massachusetts General Hospital, Harvard Medical School, Boston, MA 02114-1107, USA; 3 Neurodegenerative Diseases Group, Biocruces Bizkaia Health Research Institute, 48903 Barakaldo, Spain; 4 Department of Neurology, Cruces University Hospital, 48903 Barakaldo, Spain; 5 Ophthalmology Department, Cruces University Hospital, 48903 Barakaldo, Spain; 6 Department of Neurosciences, University of the Basque Country (UPV/EHU), 48940 Leioa, Spain; 7 IKERBASQUE, The Basque Foundation for Science, 48009 Bilbao, Spain

**Keywords:** synucleinopathies, Lewy bodies diseases, Parkinson’s disease, E46K-SNCA, cognition, nonmotor, clustering analysis, multimodal MRI

## Abstract

Background: We aimed to characterize subtypes of synucleinopathies using a clustering approach based on cognitive and other nonmotor data and to explore structural and functional magnetic resonance imaging (MRI) brain differences between identified clusters. Methods: Sixty-two patients (*n* = 6 E46K-SNCA, *n* = 8 dementia with Lewy bodies (DLB) and *n* = 48 idiopathic Parkinson’s disease (PD)) and 37 normal controls underwent nonmotor evaluation with extensive cognitive assessment. Hierarchical cluster analysis (HCA) was performed on patients’ samples based on nonmotor variables. T1, diffusion-weighted, and resting-state functional MRI data were acquired. Whole-brain comparisons were performed. Results: HCA revealed two subtypes, the mild subtype (*n* = 29) and the severe subtype (*n* = 33). The mild subtype patients were slightly impaired in some nonmotor domains (fatigue, depression, olfaction, and orthostatic hypotension) with no detectable cognitive impairment; the severe subtype patients (PD patients, all DLB, and the symptomatic E46K-SNCA carriers) were severely impaired in motor and nonmotor domains with marked cognitive, visual and bradykinesia alterations. Multimodal MRI analyses suggested that the severe subtype exhibits widespread brain alterations in both structure and function, whereas the mild subtype shows relatively mild disruptions in occipital brain structure and function. Conclusions: These findings support the potential value of incorporating an extensive nonmotor evaluation to characterize specific clinical patterns and brain degeneration patterns of synucleinopathies.

## 1. Introduction

The interest in the cognition of Parkinson’s disease (PD) has grown considerably over the years [1,2]. Only 15% of PD patients remain cognitively intact in the long-term [3]; although 20% of PD patients will fulfill criteria for mild cognitive impairment (MCI) [4], up to 46% of patients with PD and MCI will progress to dementia in 10 years [5,6]. These alterations in cognition also vary depending on whether the PD case is idiopathic or genetic. Apart from cognitive dysfunction, PD is a complex and heterogenic disease in terms of clinical presentation. The heterogeneity of this disease has led to increased interest in patient subtyping based on motor and nonmotor manifestations, and it is only now starting to be understood [7,8]. What it is clear is that good subtyping at baseline study selection is crucial for future clinical trial designs. Data-driven approaches and cross-sectional studies [8,9,10,11,12] have hypothesized that there are different PD subtypes. Few studies on PD subtypes consider a complete assessment of nonmotor symptoms as well as an extensive cognitive evaluation. A recent study [10] found four clusters replicated in two independent cohorts (Tracking Parkinson’s and Discovery) of newly diagnosed patients with PD. However, in this recent study [10], cognition was only recorded by using the Montreal Cognitive Assessment (MoCA) adjusted for education years and by using semantic verbal fluency (animals). The classification of MCI in PD (PDMCI) as established by the Movement Disorders Society (MDS) Task Force criteria [4] defines two levels of assessment. Level I is based on a global cognitive scale, whereas Level II is based on a comprehensive assessment that includes two tests per cognitive domain. It is therefore important to ascertain which subtypes of PD exist based on motor and nonmotor symptoms, but one must bear in mind the specific differences in the entire cognitive profile based on Level II of the PDMCI.

Gray matter (GM) and white matter (WM) data obtained from magnetic resonance imaging (MRI) also helped classify PD patients with cluster analysis [13,14,15,16]. However, there are very few studies investigating PD subtyping based on whole-brain resting-state functional connectivity (FC). A recent study showed heterogeneous subtypes of PD patients in which depression symptoms had a considerable impact on brain damage affecting FC in patients [17]. In addition, in some PD patients with more aggressive phenotypes, cognitive impairment occurred in early phases of the disease, when, neurobiologically, the cause of cognitive fluctuations is likely to originate from alterations in the functional network rather than from structural alterations [18]. These patients with aggressive phenotypes of PD share clinical and pathological characteristics with two less common diffuse synucleinopathies: PD associated with the E46K mutation of the alpha-synuclein gene (E46K- SNCA) [19,20] and dementia with Lewy bodies (DLB) [21,22,23]. In 2004, our group described a mutation in the SNCA gene (E46K substitution in SNCA) in a family with autosomal dominant PD and DLB [20]. The mutation produced glutamic acid substitution with lysine in position 46 of the alpha-synuclein gene (E46K-SNCA). Mutation carriers showed extensive Lewy bodies and Lewy neurites in subcortical and cortical structures that met the pathological criteria for DLB, and it induced a Lewy body disease in the brain with an aggressive clinical phenotype, including motor and nonmotor alterations (mood disorders, early cognitive impairment, and visuospatial disorders). In fact, one of the strengths of our work is that we tried to investigate the brain mechanisms of synucleinopathies while differentiating between specific clinical subtypes and while using an excellent genetic model of idiopathic PD. We sought to know whether, in addition to a different clinical profile, the described brain alterations are specific to clinical subtypes or are shared across different subtypes.

Therefore, we aimed to characterize patients using a clustering approach based on cognitive and other nonmotor data, and we involved idiopathic PD patients, E46K-SNCA carriers, and DLB patients. Additionally, we explored whole-brain structural (T1 and diffusion-weighted) and resting-state functional differences between the clusters identified and compared them to normal controls. A good definition of these clusters will be important for understanding the etiology of the disease, for discovering biomarkers related to prognosis, and even for making different interventions that are much more appropriate to the clinical subtype and to the specific cognitive profile.

## 2. Materials and Methods

### 2.1. Participants

Sixty-two patients (6 E46K-SNCA, 8 DLB, 48 idiopathic PD) and thirty-seven normal control patients were included in this study. Participants were recruited at Cruces University Hospital (Department of Neurology) and at the PD Biscay Association (ASPARBI). Patients with idiopathic PD fulfilled the Parkinson’s UK Brain Bank criteria for the diagnosis of PD, and patients with DLB fulfilled the diagnosis of probable DLB by revised criteria for the clinical diagnosis of DLB. All patients were evaluated in on-medication states (more information in [24]). For the MRI part of the study, further exclusion criteria included problems with the pre-processing of MRI data or with whole-group analysis. From the initial sample of 62 patients and 37 controls, one patient refused to attend MRI acquisition, four patients were excluded from the T1-weighted structural MRI analysis, and one control was excluded from the resting-state functional MRI analysis. Hence, MRI analyses were carried out on 57 patients and 36 controls. No significant differences were found between the included and the excluded patients.

### 2.2. Demographic and PD-Related Features Assessment

Age, sex, and years of education were registered for all participants. PD-related features were also recorded (see Supplementary Material Table S1).

### 2.3. Nonmotor Assessment

#### 2.3.1. Cognitive and Clinical Assessment 

Cognition was assessed with MoCA as a test of cognitive screening and with a broad range of standardized neuropsychological tests. Five cognitive domains with the tests recommended by the MDS criteria for diagnosis of PDMCI (Level II) were created [4]: attention and working memory, executive functions, language, memory, and visuospatial functions. Single-domain MCI (SDMCI) was categorized as when abnormalities in two tests within a single cognitive domain were present, and multiple-domain MCI (MDMCI) was categorized as when abnormalities in at least one test in two or more cognitive domains were present. Patients who did not meet these specific criteria were classified as noMCI. In addition, processing speed and theory of mind were measured. Depression, apathy, fatigue symptoms, quality of life, and activities of daily living were also recorded (see Supplementary Material Table S1). 

#### 2.3.2. Dysautonomia, Olfaction, and Visual Assessment 

Orthostatic hypotension (OHT), blood pressure recovery time (PRT), heart rate response (variability) to deep breathing (HRVdb) (more information in [25] and Supplementary Material Table S1), olfaction (BSIT), visual functioning (VFQ-25), binocular low-contrast visual acuity (LCVA), and photopic contrast sensitivity (PCS) were measured (more information in [24] and Supplementary Material Table S1). 

### 2.4. Selection of Variables and Clustering Analysis

To simplify the model and reduce the bias in clustering algorithms due to highly correlated variables, we used a random forest feature selection technique. This method allowed us to select the combination of cognitive and other nonmotor variables that best differentiated between patients and controls. These variables were chosen for hierarchical clustering analysis (HCA): attention and working memory, executive functions, language, memory, visuospatial functions, and processing speed (cognition); GDS-15 (depressive symptoms); OHT, PRT, and HRVdb (dysautonomia); BSIT (olfaction); LCVA and PCS (visual). Variables were converted to z scores to conduct the HCA, which was performed including only patients (synucleinopathies). Features related to the disease were not included in the HCA. The HCA was based on a bottom-up approach. A complete linkage criterion was used to minimize the maximum distance between observations of pairs of clusters. Using the silhouette method, we found k = 2 clusters to be the optimal partition. For both clusters, we obtained the average z score of each variable to perform the HCA. Scikit-Learning running under Python version 3.6.5 was employed. 

### 2.5. Neuroimaging Preprocessing and Analysis 

Structural and functional neuroimaging brain data were acquired using a 3–T MRI scanner (Philips Achieva TX, USA) at OSATEK, Hospital of Galdakao. All sequences were acquired during a single session. The neuroimaging acquisition parameters’ descriptions are included in Supplementary Material 1 (S2: Neuroimage acquisition).

#### 2.5.1. Structural and Diffusion MRI Preprocessing and Analysis

Voxel-based morphometry (VBM) [26] preprocessing was carried out using the FMRIB Software Library (FSL) tools version 6.0 [27] (for more information, see Supplementary Material 1, S3: Structural, diffusion and resting-state functional MRI preprocessing). Whole-brain GM analyses were performed with a randomized tool (5000 permutations) and with the threshold-free cluster enhancement (TFCE) methodology. The statistical threshold for analysis was set at *p* < 0.05 and was corrected for multiple comparisons by using the family-wise error (FWE) rate and by including sex, age, years of education, and total intracranial volume (TIV) as covariates.

The FSL [27] version 6.0 and Brain Extraction Tool (BET) [28] was used for the preprocessing and analysis of diffusion data (for more information, see Supplementary Material 1, S3: Structural, diffusion and resting-state functional MRI preprocessing). Whole-brain WM analyses were performed with a randomized tool (5000 permutations) and with TFCE methodology. The statistical threshold for analysis was set at *p* < *0*.05 and was corrected for multiple comparisons by using the FWE rate and by including sex, age, and years of education as covariates.

#### 2.5.2. Resting-State Functional MRI Preprocessing and Analysis

Resting-state functional MRI (rs-fMRI) data were preprocessed (band-pass filtering was performed with a frequency window of 0.008 to 0.09 Hz [29]) and analyzed using Conn Functional Connectivity Toolbox version 20.0 [30] (for more information, see Supplementary Material 1 S3: Structural, diffusion and resting-state functional MRI preprocessing). FC differences were assessed with the region of interest (ROI)-to-ROI methodology, and the statistical threshold was set at *p* < *0*.05 and was corrected corrected for multiple comparisons by using false discovery rate (FDR) and by including sex, age, and years of education as covariates. In addition, LEDD data were also included in the FC analysis as covariates [31]. The ROIs selected for FC analysis were based on the FC atlas networks of the CONN toolbox: Default Mode Network, Sensorimotor, Visual, Salience/Cingulo-Opercular, Dorsal Attention, Fronto Parietal/Central Executive, Language, and Cerebellar. For specific network information, see CONN network cortical ROIs HCP-ICA [30].

### 2.6. Data Analysis

The normality of the data was tested using the Shapiro–Wilk test. Significant differences in demographic, cognitive, clinical, and other nonmotor variables were assessed with analysis of variance (ANOVA) and LSD post-hoc tests. PD-related features’ differences between identified clusters were assessed with a two-tailed *t*-test. Categorical data were analyzed with a chi-squared (χ2) test. Statistical analyses were performed using the statistical package SPSS program (IBM SPSS Statistics v 27.0).

## 3. Results

### 3.1. Subtypes of Synucleinopathies Based on Cluster Analysis

**Figure 1:**
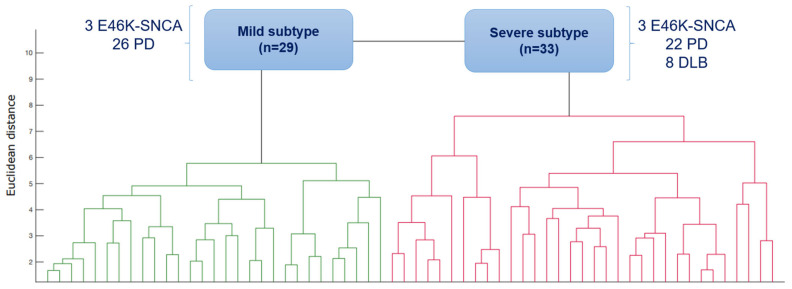
Dendrogram of patients clustered according to nonmotor data. Abbreviations: E46K-SNCA = E46K mutation of the alpha-synuclein gene; PD = Parkinson’s disease; DLB = Dementia with Lewy bodies.

We identified two cluster subtypes in synucleinopathies (see Figure 1). Twenty-six idiopathic PD patients and the three less affected and younger E46K-SNCA carriers comprised the mild subtype (*n* = 29), whereas all DLB patients (*n* = 8), 22 idiopathic PD patients and the three more affected E46K-SNCA carriers comprised the severe subtype (*n* = 33).

**Figure 2:**
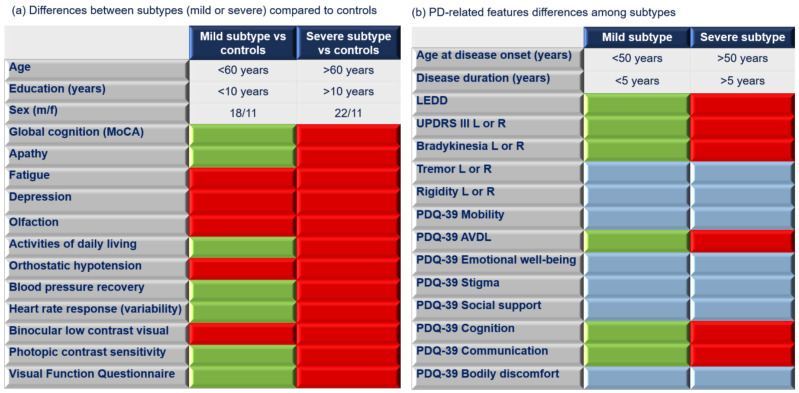
Demographic, nonmotor, and PD-related features of subtypes. (**a**) Graphical representation of demographic and nonmotor differences between the two identified clusters compared to controls. Red color indicates significantly (*p* < 0.05) lower scores compared to controls; green color indicates no significant (*p* > 0.05) differences compared to controls. (**b**) Graphical representation of PD-related features’ differences between clusters identified (mild subtype vs. severe subtype). Red color indicates significantly (*p* < 0.05) lower scores from the severe subtype compared to the mild subtype; green color indicates significantly (*p* < 0.05) higher scores from the mild subtype compared to the severe subtype; blue color indicates no significant (*p* > 0.05) differences between clusters. Abbreviations: m = male; f = female; MoCA = Montreal Cognitive Assessment; LEDD = Levodopa Equivalent Daily Dose; UPDRS = Unified Parkinson’s Disease Rating Scale; L = left; R = right; PDQ-39 = Parkinson’s Disease Questionnaire 39 items; AVDL = Activities of Daily Living.

The mild subtype patients showed no significant differences in demographic variables when compared to normal controls. Mild subtype patients scored significantly higher in orthostatic hypotension, fatigue, and depression, and they had lower scores in visual acuity and olfaction than controls (see Figure 2). Additionally, mild subtype patients were younger, had more education years, were younger at disease onset, and scored significantly higher in all cognitive domains, dysautonomia, and PD-related features as well as in ADL compared to the severe subtype. The severe subtype patients showed significantly higher ages and fewer years of education compared to controls and to mild subtype. Additionally, severe subtype patients showed more nonmotor alterations compared to controls and had more severe motor symptoms (UPDRS III) compared to mild subtype patients. Specifically, more marked cognitive, visual and bradykinesia alterations were found. Severe subtype patients had the greatest average age of disease onset and the oldest ages, but they did not differ significantly in disease duration compared to the mild subtype. The demographic, cognition, PD-related features, dysautonomia, visual, and clinical differences between all patients (mild subtype and severe subtype) and controls are shown in Table 1. Specifically, in demographic variables, controls showed significant differences from the severe subtype patients in age and years of education; therefore, these variables were used as covariates in neuroimaging analysis.

### 3.2. Cognitive Profile of Subtypes According to PDMCI Level II Criteria

**Figure 3:**
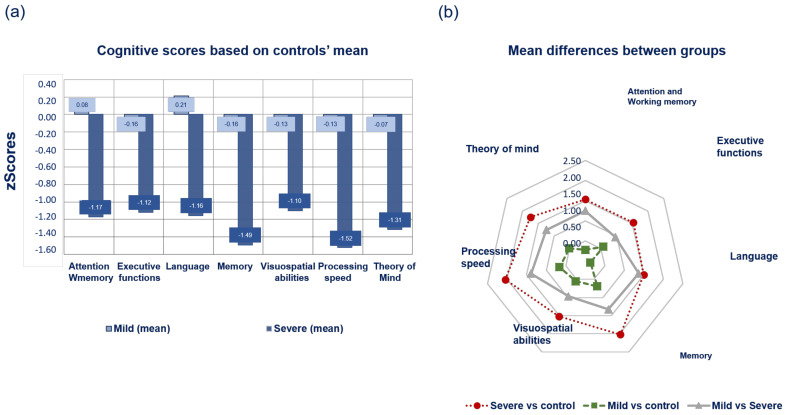
Cognitive profile of subtypes. (**a**) Means of cognitive domains of patients compared to controls (z = 0 is the baseline according to the mean of controls). Data are presented as z scores, and in all cases, lower z scores indicate worse performance. (**b**) Mean differences of ANOVA post-hoc analysis of the sample between subtypes and controls. Data are presented as z scores, and in all cases, lower z scores indicate less prominent differences between groups.

Mild subtype patients scored lower in executive functions, memory, visuospatial abilities, processing speed, and theory of mind, and they scored higher in language compared to controls and higher in all cognitive domains compared to severe subtype patients (see Figure 3a). Moreover, mean differences between mild subtype patients and controls were higher in the memory, executive functions, and theory of mind domains (see Figure 3b). Severe subtype patients scored lower than mild subtype patients and controls in all domains. Memory, language, processing speed, and theory of mind were the domains with the lowest means based on z scores (see Figure 3a) and based on the mean differences between groups (see Figure 3b). 

The percentage of MCI in each subtype showed two patterns. The severe subtype comprised 94% of patients with MDMCI (*n* = 31), 3% with noMCI (*n* = 1), and 3% of patients with SDMCI (*n* = 1, memory domain), whereas in the mild subtype, 86% of patients were categorized as noMCI (*n* = 25), 10% as MDMCI (*n* = 3), and 4% of patients as SDMCI (*n* = 1, executive functions domain).

### 3.3. Structural and Functional Brain Degeneration in Synucleinopathies Based on Clusters

**Figure 4:**
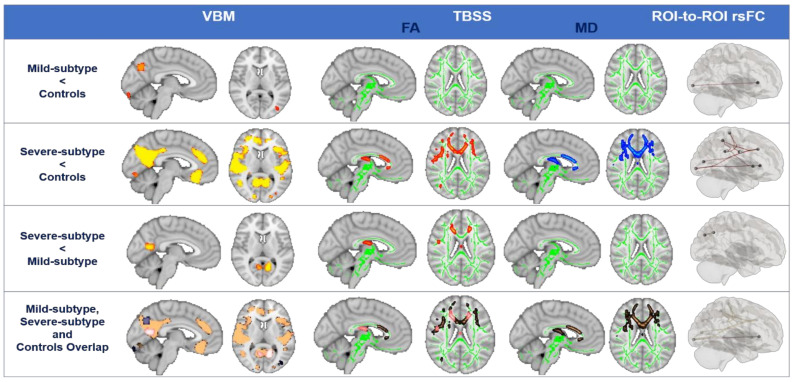
Structural and functional brain degeneration in synucleinopathies compared to controls. Voxel-based morphometry (VBM), tract-based spatial statistics (TBSS), and region of interest (ROI)-to-ROI functional connectivity (FC) analyses of the sample. VBM analysis: regions with less gray matter volume are shown in red–yellow shades (*p* < 0.05, FWE-corrected). Results were adjusted by age, sex, years of education, and TIV. TBSS analysis: regions with less FA are shown in red–yellow shades, and those with higher MD are shown in blue to light blue shades. FA skeleton mask (green) (*p* < 0.05, FWE-corrected). Results were adjusted by age, sex, and years of education. FC analysis: regions with less FC are shown in red (*p* < 0.05, FDR-corrected). Results were adjusted by age, sex, years of education, TIV, and Levodopa Equivalent Daily Dose (LEDD). Abbreviations: FA = fractional anisotropy; MD = medial diffusivity.

Structural and functional brain results in patients based on clusters are shown in Table A1, Table A2 and Table A3 in the Appendix A and in Figure 4. Mild subtype patients exhibited marked left occipital and precuneus GM alterations and lower FCs between visual occipital and language networks (left inferior frontal gyrus) compared to controls, but they exhibited no significant WM alterations compared to controls. Severe subtype patients showed bilateral frontotemporal (including left hippocampus) and occipital GM alterations compared to controls as well as lower FA WMs in bilateral anterior thalamic radiation and right longitudinal fasciculus and higher MD WMs in bilateral anterior thalamic radiation, left cingulum, right longitudinal, and body of corpus callosum compared to controls. In addition, visual, dorsal-attentional, salience, language, and sensorimotor FC alterations compared to controls were found. When both subtypes were compared, similar GM patterns were found when we compared the mild subtype patients with normal controls. Severe subtype patients exhibited GM alterations in the left hippocampus, right fronto-orbital, left occipital, and left precuneus compared to those with the mild subtype. WM FA differences were also found between subtypes; specifically, the severe subtype presented lower FA WM in right anterior thalamic radiation, left cingulum, right longitudinal, and body of corpus callosum compared to the mild subtype. Finally, marked lower FC in the posterior-lateral DMN was found in severe subtype patients compared to those with the mild subtype.

## 4. Discussion

To the best of our knowledge, this is the first study that investigated the heterogeneity of synucleinopathies, classified them using clustering analysis, and included an extensive neuropsychological battery of tests and a broad spectrum of other nonmotor features. Additionally, we explored the specific cognitive profiles of patients based on PDMCI Level II criteria, and we investigated with multimodal MRI the brain’s structural and functional alterations behind these subtypes. The main findings were as follows: (a) clustering analysis using nonmotor features defined two subtypes with very different clinical profiles; (b) the severe subtype was characterized by motor and nonmotor alterations with marked cognitive impairment, whereas the mild subtype only presented nonmotor alterations in visual acuity, olfaction, dysautonomia, fatigue, and depression; (c) the severe subtype showed MDMCI with memory, processing speed, language, and theory of mind as the most affected domains; (d) older E46K-SNCA carriers and DLB patients showed MDMCI with similar cognitive profiles and marked visuospatial alterations, whereas PD patients showed heterogeneity with the PD severe subtype showing MDMCI and amnestic SDMCI, and the PD mild subtype showing noMCI and executive SMDMCI; (e) the severe subtype revealed widespread disruptions in function and structure in the fronto-temporal and occipital areas, whereas the mild subtype showed relatively mild brain abnormalities that were mainly in occipital areas.

### 4.1. Distinct Clinically Relevant Patterns in Synucleinopathies

In this study, we considered several nonmotor features together, and we revealed two clinically relevant subtypes that were most associated with specific nonmotor symptoms. The main results of this study are in line with previous studies in the literature. According to Van Rooden [8], the majority of studies reported two clusters with very similar profiles in terms of age-at-onset and rates of disease progression. In fact, a recent study [32] also showed two clusters of PD patients, which were mild-motor predominant and severe cluster (diffuse malignant); apart from motor and nonmotor symptoms [33], MRI studies also showed two subgroups of PD patients [13] with differences in cortical atrophy. However, other studies showed three or four different clusters [14,34]. In the study by Mu and colleagues [34], four clusters were identified: mild, nonmotor-dominant, motor-dominant, and severe. In another structural MRI study [14], three subtypes of PD were found with prominent differences in GM patterns and little WM involvement. Finally, regarding rs-fMRI, one study evaluated the FC of networks in PD, and, as in our study, two significant patterns were found: “motor-related pattern” and “depression-related pattern”. 

It is widely known that nonmotor symptoms of PD may have a greater impact on quality of life than motor features, and they may even precede overt signs and symptoms of motor disturbances [35,36]. In our study, severe subtype patients exhibited a clear deterioration of their cognitive and clinical profiles with alterations in motor features, the more marked symptom being bradykinesia alterations. Interestingly, we observed in the mild subtype mild motor alterations but significant disturbances in olfaction (hyposmia), dysautonomia (orthostatic hypotension), visual acuity, fatigue, and depression. In addition, these results were found when patients did not have significantly different disease durations, although the mean age and age at onset were lower in the mild subtype. Therefore, and according to a similar study [14], these findings reinforce the idea that later onset of the disease is associated with rapid disease progression [37]. Furthermore, recent reviews in animal models [38] highlight the importance of the presence of Lewy bodies (made predominantly of alpha-synuclein) in relation to motor and nonmotor symptomatology, such as olfactory dysfunction, anxiety, depression, and cognitive dysfunction.

One hypothesis about the neuropathological stages of PD suggests that Lewy body pathology in the nigrostriatal system only develops after lower brainstem areas and the olfactory system are affected [39,40]. In our study, one of the prominent alterations of the mild subtype was related to olfaction. Therefore, this could imply that hyposmia is present from the start of the disease. Recent studies suggest that hyposmia may be an early preclinical sign, is related to an increased risk to develop overt PD in asymptomatic first-degree relatives of PD patients, and is a risk marker for the general population [41,42]. Orthostatic hypotension is a frequent nonmotor symptom in alpha-synucleinopathies including PD and DLB, and it occurs in 20–50% of PD cases and 30–70% of DLB cases [43,44]. This alteration was also found in mild subtype patients. In fact, a previous study of our group showed that dysautonomia was also related to neuropsychological performance and to depression and apathy symptoms in Lewy body diseases [25]. LCVA (low-contrast visual acuity) was another alteration in the mild subtype. Visual disturbances in general are relatively common in PD, with some studies reporting that up to 78% of patients are affected, including reduced contrast sensitivity, impaired color discrimination, convergence insufficiency, and dry eye syndrome [45]. In a previous study, we identified that primary visual function was significantly worse in patients than in controls, mainly expressed as LCVA, which was severely impaired in the E46K–SNCA and DLB patients and moderately impaired in idiopathic PD patients [46]. Depression could appear in the prodromal phase of PD and was shown to nearly double an individual’s risk of subsequently developing PD [47]. In addition, fatigue often appears in the early motor stage, and it is identified by persons with PD as one of their most disabling symptoms with the greatest impact on their quality of life [47,48]. Fatigue can be present in 50% of people with PD. In some patients, fatigue is also related to depression and autonomic symptoms; however, whereas depression and its brain correlates are highly investigated in PD, very little is known about the mechanisms of fatigue in PD. Finally, these alterations (hyposmia, orthostatic hypotension, visual deficits, depression, and fatigue) are usually related to future cognitive decline in PD. Thus, the five nonmotor alterations identified in the mild subtype of this study mark the increasingly important role of nonmotor symptoms in synucleinopathies’ heterogeneity and the importance to take steps toward early identification and subtype-specific treatment packages.

### 4.2. Distinct Cognitive Profiles in Synucleinopathies

The severe subtype was 94% composed of patients with MDMCI, whereas in the mild subtype, 86% of patients were categorized as noMCI, 10% as MDMCI, and 4% of patients as SDMCI (executive functions domain). Interestingly, mild cognitive impairment is also a common in early PD, and the main feature is usually impairment in executive functions [47]. One study showed that the worst cognitive domains among converters to PD dementia compared with nonconverters to PD dementia were the executive function/working memory domains [49]. In addition, the severe subtype group, apart from MDMCI, also showed more bradykinetic symptoms; patients with those cognitive alterations plus patients with the bradykinetic-rigid form of PD are more at risk of developing dementia [47].

Additionally, the results showed that young E46K-SNCA carriers’ cognitive profiles were very similar to the controls’ cognitive profiles, demonstrating noMCI (100% of noMCI) in the sample. However, all aggressive E46K-SNCA carriers exhibited MDMCI, as all DLB patients did, with more marked visuospatial alterations and even lower scores in all domains evaluated compared to the DLB group. This concept is related to previous studies in the literature suggesting that early visual-cognitive dysfunction is one of the main predictors for the development of cognitive disability in PD [50].

Apart from PD-MCI Level II criteria domains, other cognitive domains, such as processing speed or theory of mind, are frequently altered in PD. When we compared the cognitive profiles of the patients, the mild subtype group scored lower in executive functions, memory, visuospatial abilities, processing speed, and theory of mind, and they scored higher in language compared to controls. One study revealed that the unique domain that did not worsen among converters compared with non-converters PD dementia was the language domain [49]. However, other studies showed that semantic fluency was shown to be a predictor of dementia in PD [50]. The severe subtype group scored lower than the mild subtype group and controls in all domains with the lowest mean scores, those being memory, language, processing speed, and theory of mind. Processing speed is a cognitive domain that is not included in the Level II of PD-MCI, but processing speed disturbances are widely present in PD, and they usually affect daily living activities [51]. In fact, cognitive alterations in processing speed are correlated with fatigue in PD [52] and clinical symptoms which usually appear in the early phases of the disease. In the case of social cognition and theory of mind, one recent study [53] suggested that deficits in the DMN may be contributing to theory of mind deficits in amnestic MCI, highlighting the importance of including measures of social cognition in the clinical routine to detect MCI. Moreover, neurodegenerative disorders frequently present with social cognitive, memory, and executive impairments, offering an opportunity to explore the intersection between theory of mind and cognitive functions. Interestingly, when executive function performance is controlled for, specific WM alterations were found, implying a domain-specific theory of mind impairment in PD [54].

### 4.3. Brain Degeneration in Synucleinopathies Based on Neuroimaging: Towards a Pathophysiological Explanation

Severe subtype patients showed bilateral frontotemporal and occipital GM and WM alterations compared to controls as well as visual, dorsal attentional, salience, language, and sensorimotor FC alterations. However, the mild subtype only showed marked left occipital and precuneus GM alterations, no significant WM alterations, and lower FC between visual occipital and language networks (left inferior frontal gyrus) compared to normal controls. When both subtypes were compared, the severe subtype group exhibited GM alterations in the left hippocampus, right fronto-orbital, left occipital, and left precuneus compared to the mild subtype group as well as lower FA WM in the right anterior thalamic radiation, left cingulum, right longitudinal fasciculus, and body of corpus callosum compared to the mild subtype group. Finally, markedly lower FC in the posterior-lateral DMN was found in severe subtype patients compared to mild subtype patients.

It is also worth noting that even though both WM and GM contributed to explain the different subtypes, GM degeneration patterns were more relevant in the characterization of PD groups than WM alterations. The majority of whole-brain studies evidenced the involvement of fronto-occipital WM tracts [14], such as the corpus callosum, cingulum, and other major association tracts in PD patients with MCI [16,55,56]. Indeed, in this study, WM FA differences were found between subtypes specifically in the cingulum, corpus callosum, and longitudinal fasciculus, suggesting that WM impairment in PD might be a sign preceding the neuronal loss in associated GM areas.

What it is clear is that there are extensive atrophic changes as well as FC changes in bilateral fronto-temporo-occipital regions in the severe subtype, whereas marked fronto-occipital alterations appear in the mild subtype. This dissociation in the severe subtype could be related not only to cognitive impairment but also to other nonmotor symptoms. The frontal and medial occipital lobes are speculated to be associated with nonmotor symptoms [57], and in previous studies in the literature, prefrontal disturbances were also associated with depression [17] and hyposmia [58] in PD. In addition, mild subtype patients showed depressive symptoms. A recent study indicated that the presence of depressive symptoms in patients with early PD is associated with a higher risk of progression to MCI, and that early depression may reflect subsequent cortical atrophy [59]. Moreover, visual disturbances were also found in the mild subtype; given that there are important nodes associated with visual information processing in occipital region, this suggests that abnormal FC in occipital lobe underpins both primary visual perceptual and intrinsic visual integration. In fact, visual disturbances are associated with more pronounced cognitive deterioration in PD [24]. Therefore, incorporating resting-state FC measures other than structure into these studies involves moving one step closer to multimodal MRI approaches. 

However, some limitations of this study should be noted. First, our data-driven cluster analysis needs to be validated in independent cohorts. Second, longitudinal studies on large cohorts of patients will be crucial to confirm our results and to accurately follow brain modifications from synucleinopathies’ progression. Third, although the inclusion of synucleinopathies and, specifically, E46K mutation is one of the highlights of the present work, the sample size was small, and future studies with larger samples are needed to deeply explore the structural and functional brain differences across stages of synucleinopathies.

## 5. Conclusions

The current study opens new perspectives by being the first to assess brain network degeneration based on cognition and other nonmotor features in synucleinopathies by using a multimodal neuroimaging approach. Moreover, it is important to remark that SNCA-linked mutations are limited to specific families and series worldwide, and that their study is a unique opportunity to improve our understanding of different synucleinopathies’ phenotypes. These results shed light on different phenotypes in synucleinopathies, which not only differ in cognitive performance but also in other nonmotor symptoms (visual acuity, olfaction, dysautonomia, fatigue, and depression) and brain degeneration. Thus, the hypothesis of distinct disease courses and treatments is supported.

## Figures and Tables

**Table 1:** Demographic, PD-related, and nonmotor characteristics of the sample.

Variables	Synucleinopathies (*n*:62)		Controls (*n*:37)		Comparisons	
	Mild (*n*:29)	Severe (*n*:33)		Among Subtypes (*p* Value)	Subtypes vs. Controls (*p* Value)	
	Mean (SD)	Mean (SD)	Mean (SD)		Mild vs. Controls	Severe vs. Controls
Demographics						
Age, years	53.60 (8.78)	67.55 (6.73)	53.48 (12.74)	**0.000**	0.960	**0.000**
Sex (m/f)	18/11	22/11	21/16	0.793	0.802	0.465
Education, years	13.24 (3.45)	8.61 (4.03)	13.84 (5.05)	**0.000**	0.577	**0.000**
Cognition						
MoCA	26.51 (1.76)	20.54 (5.02)	27.24 (3.14)	**0.000**	0.419	**0.000**
Attention and WM	0.44 (0.52)	−0.81 (0.56)	0.36 (0.78)	**0.000**	0.601	**0.000**
Memory	0.37 (0.62)	−0.96 (0.64)	0.53 (0.58)	**0.000**	0.302	**0.000**
Executive functions	0.39 (0.56)	−0.92 (0.87)	0.48 (0.44)	**0.000**	0.375	**0.000**
Language	0.41 (0.49)	−0.94 (0.48)	0.46 (0.82)	**0.000**	0.161	**0.000**
Visuospatial abilities	0.29 (0.55)	−0.76 (0.87)	0.47 (0.55)	**0.000**	0.439	**0.000**
Processing speed	0.40 (0.51)	−0.99 (0.50)	0.53 (0.80)	**0.000**	0.423	**0.000**
Theory of mind	0.37 (0.74)	−0.87 (0.96)	0.44 (0.70)	**0.000**	0.720	**0.000**
PD-related features						
Disease duration, years	5.80 (3.52)	7.85 (4.86)	-	0.078	-	-
Age of onset, years	48.34 (7.29)	59.29 (8.05)	-	**0.000**	-	-
UPDRS III R no midline	7.14 (5.27)	11.28 (3.89)	-	**0.001**	-	-
UPDRS III L no midline	8.61 (4.49)	12.21 (5.11)	-	**0.007**	-	-
Bradykinesia R	4.30 (2.83)	7.07 (1.98)	-	**0.000**	-	-
Bradykinesia L	5.52 (2.46)	7.66 (2.72)	-	**0.003**	-	-
Rigidity	2.11 (1.55)	2.93 (1.71)	-	0.066	-	-
Rigidity L	2.44 (1.58)	3.14 (1.66)	-	0.116	-	-
Tremor R	0.89 (2.23)	1.45 (1.76)	-	0.300	-	-
Tremor L	0.93 (1.47)	1.59 (1.97)	-	0.159	-	-
LEDD	618.76 (369.41)	736.22 (454.39)	-	0.295	-	-
PDQ-39 Mobility	9.40 (8.88)	11.79 (9.08)	-	0.325	-	-
PDQ-39 AVDL	4.51 (5.42)	8.10 (6.93)	-	**0.035**	-	-
PDQ-39 EW	7.11 (5.43)	6.31 (6.89)	-	0.633	-	-
PDQ-39 Stigma	2.59 (2.91)	2.20 (4.91)	-	0.725	-	-
PDQ-39 SS	1.25 (2.56)	1.59 (2.18)	-	0.609	-	-
PDQ-39 Cognition	3.25 (2.83)	5.38 (4.73)	-	**0.046**	-	-
PDQ-39 Com	1.22 (1.88)	3.20 (3.57)	-	**0.012**	-	-
PDQ-39 BD	4 (3.44)	5.41 (3.81)	-	0.152	-	-
Dysautonomia						
OHT	0.66 (0.87)	0.58 (0.84)	0.12 (0.33)	0.663	**0.012**	**0.028**
Valsalva PRT	3.69 (2.05)	7.61 (4.28)	2.64 (1.74)	**0.000**	0.177	**0.000**
HRVdb	1.01 (0.09)	0.90 (0.05)	1.03 (0.09)	**0.000**	0.334	**0.000**
Vision						
Binocular LCVA	32.93 (6.11)	18.19 (13.21)	37.10 (6.38)	**0.000**	**0.031**	**0.000**
Photopic CS	2.0 (0.11)	1.85 (0.14)	2.05 (0.13)	**0.000**	0.086	**0.000**
VFQ-25	92.22 (11.99)	84.32 (15.01)	96.08 (4.66)	**0.008**	0.187	**0.000**
Clinical						
Apathy	27.89 (5.13)	22.62 (8.61)	29.5 (4.04)	0.057	0.551	**0.004**
Fatigue	29.76 (17.25)	34.50 (16.57)	20.81 (10.96)	0.218	**0.017**	**0.000**
Depression	2.59 (2.33)	3.75 (3.52)	1.11 (1.62)	0.082	**0.023**	**0.000**
Olfaction	7.68 (2.30)	5.48 (2.61)	10.48 (1.21)	**0.000**	**0.000**	**0.000**
AVDL	7.83 (0.75)	6.19 (2.28)	8 (0)	**0.000**	0.612	**0.000**

Abbreviations: SD = standard deviation; m = male; f = female; MoCA = Montreal Cognitive Assessment; WM = working memory; UPDRS = Unified Parkinson’s Disease Rating Scale; III = motor part; R = right; L = left; LEDD = Levodopa Equivalent Daily Dose; PDQ-39 = Parkinson’s Disease Questionnaire 39 items; AVDL = Activities of Daily Living; EW = emotional wellbeing; SS = social support; Com = communication; BD = bodily discomfort; OHT = orthostatic hypotension; PRT = blood pressure recovery time; HRVdb = heart rate variability to deep breathing; LCVA = low-contrast visual acuity; CS = contrast sensitivity; VFQ-25 = Visual Functionary Questionnaire 25 items.

## Data Availability

The data supporting the findings of this study are available from the corresponding author upon reasonable request. They are not publicly available due to ethical restrictions.

## Supplementary Materials

The following supporting information can be downloaded at: https://www.mdpi.com/article/10.3390/biomedicines11020573/s1, Table S1. PD-related features and non-motor assessment; S2. Neuroimaging acquisition; S3. Structural, diffusion and resting-state functional MRI preprocessing.

## Appendix A

**Table A1:** Structural gray matter differences.

Regions	Cluster Size (Voxels)	Stats	*p* Value (FWE-Corrected)	MNI Coordinates X Y Z
Mild < Controls				
Inferior Temporooccipital Left	522	3.98	0.020	−50 −54 −10
Precuneus Left	318	4.29	0.020	−2 −70 −34
Lateral Occipital Left	223	3.96	0.031	−36 −82 −4
Occipital Pole Right	105	4.21	0.033	2 −90 −24
Severe < Controls				
Superior Temporal Right	3972	4.47	<0.001	52 −22 0
Superior Temporal Left	2408	4.56	0.005	54 −4 −12
Superior Lateral Occipital Right	2151	5.10	0.007	14 −84 24
Temporal Fusiform Right	1769	4.54	0.011	40 −34 −16
Hippocampus Left	430	4.58	0.022	−28 −32 −14
Occipital Pole Left	313	3.84	0.020	−22 −90 6
Occipital Pole Right	185	3.86	0.025	18 −88 4
Frontal Orbital Left	174	4.31	0.036	−32 30 −2
Frontal Medial Left	147	4.73	0.038	−2 30 −20
Temporal Pole Left	113	3.91	0.037	−22 6 −24
Frontal Orbital Right	106	4.76	0.032	32 28 −6
Severe < Mild				
Precuneus Left	1036	4.91	0.002	−12 −64 8
Frontal Orbital Right	82	4.34	0.035	36 30 4
Occipital Left	32	3.69	0.044	−18 −84 −14
Hippocampus Left	16	4.45	0.044	−36 −32 −6

Abbreviations: MNI = Montreal Neurological Institute.

**Table A2:** White matter fractional anisotropy and mean diffusivity differences.

Regions	Cluster Size (Voxels)	Stats	*p* Value (FWE-Corrected)	MNI Coordinates X Y Z
Severe < Controls				
FA				
Anterior Thalamic Radiation Right	13,377	3.51	<0.001	19 42 −4
Anterior Thalamic Radiation Left	366	5.51	0.007	−7 −24 14
Inferior Longitudinal Right	65	5.41	0.009	47 −21 1
Superior Longitudinal Right	56	4.39	0.010	33 4 40
Severe > Controls				
MD				
Cingulum Left	8361	3.52	0.003	−16 32 16
Anterior Thalamic Radiation Right	764	4.24	0.009	21 12 10
Superior Longitudinal Right	719	2.31	0.010	35 2 21
Anterior Thalamic Radiation Left	657	6.24	0.005	−11 −31 12
Body of Corpus Callosum	545	3.57	0.009	14 −29 28
Cingulum Left	239	4.71	0.009	−9 25 −5
Inferior Fronto-Occipital Right	127	3.43	0.010	22 51 −9
Longitudinal Right	103	2.29	0.010	25 −47 25
Severe < Mild				
FA				
Body of Corpus Callosum	1866	4.21	<0.010 *	19 −17 38
Anterior Thalamic Radiation Right	515	5.68	0.046	12 −29 13
Cingulum Left	482	4.29	<0.010 *	−16 31 21
Superior Longitudinal Right	66	3.59	<0.010 *	47 0 25

Abbreviations: MNI = Montreal Neurological Institute; FA = fractional anisotropy; MD = medial diffusivity. * Uncorrected results with *p* < 0.01.

**Table A3:** Resting-state functional connectivity differences in networks.

Seed	Target	Stats	*p* Value (FDR-Corrected)
Mild < Controls			
Visual Occipital (Visual)	IFG Left (Language)	3.75	<0.001
Severe < Controls			
IPS Left (DAN)	SMG Right (Salience)	4.19	0.024
Visual Lateral Left (Visual)	ACC (Salience)	3.93	0.028
Visual Occipital (Visual)	ACC (Salience)	3.61	0.031
Visual Occipital (Visual)	IFG Left (Language)	3.58	0.031
IPS Left (DAN)	ACC (Salience)	3.57	0.031
Sensorimotor Superior (Sensorimotor)	Sensorimotor Lateral Right (Sensorimotor)	3.55	0.031
Sensorimotor Superior (Sensorimotor)	Sensorimotor Lateral Left (Sensorimotor)	3.48	0.033
SMG Left (Salience)	aInsula Left (Salience)	3.43	0.034
SMG Left (Salience)	ACC (Salience)	3.33	0.041
Visual Occipital (Visual)	aInsula Left (Salience)	3.24	0.048
Severe < Mild			
PCC (Default Mode)	LP Left (Default Mode)	4.19	0.028

Abbreviations: DAN = Dorsal Attention Network; IPS = Intraparietal Sulcus; IFG = Inferior Frontal Gyrus; SMG = Supramarginal Gyrus; ACC = Anterior Cingulate Cortex; A = Anterior; PCC = Posterior Cingulate Cortex.
