# Supplementary material for: Brain Degeneration in Synucleinopathies Based on Analysis of Cognition and Other Nonmotor Features: A Multimodal Imaging Study
Source: Biomedicines. 2023 Feb 15;11(2):573. doi: 10.3390/biomedicines11020573 (PMC9953265; doi:10.3390/biomedicines11020573)
Supplement: Supplementary file 1 [file biomedicines-11-00573-s001.zip › biomedicines-2156068-supplementary.pdf]

Supplementary Material

Supplementary Material S1

Table S1. PD-related features and nonmotor assessment.

**PD-related features assessment**	
**Disease duration**	
PD-related features	Hoehn & Yahr Scale score<br>Unified Parkinson's Disease Rating Scale (UPDRS)
Medication	Levodopa Equivalent Daily Dose (LEDD)
**Cognitive assessment***	
Attention and working memory	Digit Span Backwards Test and Trail Making Test-A
Executive functions	Modified Wisconsin Card Sorting Test (Categories) and Clock Drawing Test order
Language	Verbal Fluency Test, which contained phonemic (P) and semantic fluency tasks (category of animals)
Memory	Hopkins Verbal Learning Test-Revised and Brief Visual Memory Test-Revised
Visuospatial functions	Benton's Judgment of Line Orientation Test (H-form) and Clock Drawing Test copy
Processing speed	Symbol Digit Modalities Test and Salthouse Perceptual Comparison Test
Theory of mind	Happé test
**Clinical assessment**	
Depression	Geriatric Depression Scale (GDS-15)
Apathy**	Lille Apathy Rating Scale (LARS)
Fatigue	Fatigue Severity Scale (FSS)
Quality of life	Parkinson Disease Questionnaire (PDQ-39)
Activities of daily living	Activities of Daily Living (AVDL)
**Dysautonomia and olfaction assessment**	
	Orthostatic hypotension (OHT) with tilt table test<br>Blood pressure recovery time (PRT) following termination of Valsalva maneuver back to baseline (seconds)<br>Heart rate response (variability) to deep breathing (HRVdb) (measured as the mean heart rate range in six respiration cycles)<br>Brief Smell Identification Test (BSIT)
**Visual assessment**	
	Visual Function Questionnaire (VFQ-25)<br>Binocular low-contrast visual acuity (LCVA) [2.5% Sloan charts at 4 meters<br>Photopic contrast sensitivity (PCS) at 1 meter with 280 lux chart luminance

* Outcome variables were converted to z scores to generate composites for each cognitive domain. The internal consistency (Cronbach's α) of each composite score was above 0.70. ** The apathy scale was only completed by 46 participants because this variable was added after the study had already started.

Supplementary Material S2

Neuroimaging Acquisition

T1-weighted image acquisition was obtained in a sagittal orientation (TR = 7.4 ms, TE = 3.4 ms, matrix size = 228 x 218mm; flip angle = 9°, FOV = 250 x 250mm, slice thickness = 1.1 mm, 300 slices, voxel size = 0.98 × 0.98 × 0.60 mm, acquisition time = 4′55′′). Diffusion-weighted images were obtained in an axial orientation in an anterior–posterior phase direction using a single-shot EPI sequence (TR = 7540 ms, TE = 76 ms, matrix size = 120 x 117mm; flip angle = 90°, FOV = 240 x 240mm, slice thickness = 2 mm, no gap, 66 slices, voxel size = 1.67 × 1.67 × 2.0 mm, acquisition time = 9′31′′) with two identical repetitions (32 uniformly distributed directions, b = 1000 s/mm2, and 1 b = 0 s/mm2). The rs-fMRI was obtained in an axial orientation in an anterior–posterior phase direction using a sequence sensitive to blood oxygen level-dependent (BOLD) contrast and a multi-slice gradient echo EPI sequence (TR = 2100 ms, TE = 27 ms, matrix size = 80x79mm, flip angle = 80°, FOV = 240x240mm, slice thickness = 3 mm, 214 scans, voxel size = 3.00 × 3.00 × 3.00 mm, acquisition time = 7′40′′).

Supplementary Material S3

Structural, Diffusion, and Resting-State Functional MRI Preprocessing

1. Structural MRI preprocessing—Voxel-based morphometry with FSL: First, a study-specific template was created so all of the images could be registered in the same stereotactic space (spatial normalization). Then, the GM images were affine registered to the GM MNI-152 template and averaged to create an affine GM template. Next, the GM images were re-registered to this affine GM template using non-linear registration and averaged to create a study-specific, non-linear GM template in standard space. Second, individual GM images were registered non-linearly to the study-specific template. After normalization, the resulting GM images were modulated by multiplying them by Jacobian determinants to correct for volume change induced by the nonlinear spatial normalization. Then, the images were smoothed with a sigma of 3.5 mm (8 mm FWHM).

2. Diffusion MRI preprocessing—Tract-based spatial statistics with FSL: First, each participant's images were concatenated and radiologically oriented. Next, data were corrected for head motion and eddy currents, brain extraction was performed using BET (Brain Extraction Tool), and the diffusion gradients (bvecs) were rotated to be corrected accordingly (Jones and Cercignani, 2010) [28]. Then, fractional anisotropy (FA) and mean diffusivity (MD) were obtained by fitting a tensor model to the raw diffusion data using FDT (DTIFIT). Afterward, voxelwise statistical analysis of the data was carried out using Tract-Based Spatial Statistics (TBSS) (Smith et al., 2004) [27]. The FNIRT tool was used to align all subjects' FA data into a common space by combining the non-linear transform to the target FA image with the affine transform from that target to MNI152 space. The mean FA image was created using a threshold of 0.2 and thinned to create a mean FA skeleton, which represented the centers of all tracts common to the group. Each participant's aligned FA data were projected onto this skeleton, and the resulting data were fed into voxelwise cross-subject statistics. The "tbss non FA" script from TBSS was used to analyze MD data. This applies the original non-lineal registration to the MD data, merges all subjects warped MD data into a 4D file, projects this onto the original mean FA skeleton, and then creates the 4D projected data.

3. Resting-state functional MRI preprocessing—ROI-to-ROI with CONN: First, each subject's 214 functional images were realigned and unwrapped, slice-timing corrected, coregistered with structural data, and spatially normalized into the standard MNI space. Then, outliers were detected (with ART-based scrubbing), and finally, images were smoothed using a Gaussian kernel of 8 mm FWMH. All preprocessing steps were conducted using a default preprocessing pipeline for volume-based analysis (to MNI-space). As recommended, band-pass filtering was performed with a frequency window of 0.008 to 0.09 Hz (Weissenbacher et al., 2009) [29]. Then, structural data were segmented into GM, WM, and cerebrospinal fluid data, and they were then normalized in the same default preprocessing pipeline (Whitfield-Gabrieli and Nieto-Castanon, 2012) [30].
